# Enhanced osteogenesis of hydroxyapatite scaffolds by coating with BMP-2-loaded short polylactide nanofiber: a new drug loading method for porous scaffolds

**DOI:** 10.1093/rb/rbz040

**Published:** 2019-11-07

**Authors:** Taotao Xu, Luyao Sheng, Lei He, Jie Weng, Ke Duan

**Affiliations:** 1 Key Laboratory of Advanced Technologies of Materials (MOE), School of Materials Science and Engineering, Southwest Jiaotong University, Chengdu 610031, China; 2 Sichuan Provincial Laboratory of Orthopaedic Engineering, Department of Bone and Joint Surgery, Affiliated Hospital of Southwest Medical University, Luzhou 646000, China

**Keywords:** short fibers, surface modification, drug release, hydroxyapatite, polylactide

## Abstract

Porous hydroxyapatite (HA) is widely used in porous forms to assist bone defect healing. However, further improvements in biological functions are desired for meeting complex clinical situations such as impaired bone regeneration in poor bone stock. The extracellular matrix (ECM) of human tissues is characterized by nanofibrous structures and a variety of signal molecules. Emulating these characteristics are expected to create a favorable microenvironment for cells and simultaneously allow release of osteogenic molecules. In this study, short polylactide fibers containing BMP-2 were prepared by electrospinning and coated on porous HA scaffolds. The coating did not affect porosity or pore interconnectivity of the scaffold but improved its compressive strength markedly. This fiber coating produced burst BMP-2 release in 1 day followed by a linear release for 24 days. The coating had a significantly lower rat calvarial osteoblasts (RCOBs) adhesion (vs. uncoated scaffold) but allowed normal proliferation subsequently. Bone marrow stem cells (MSCs) on the coated scaffolds expressed a significantly increased alkaline phosphatase activity relative to the uncoated ones. After implantation in canine dorsal muscles, the coated scaffolds formed significantly more new bone at Weeks 4 and 12, and more blood vessels at Week 12. This method offers a new option for drug delivery systems.

## Introduction

Hydroxyapatite [HA; Ca_10_(OH)_2_(PO_4_)_6_] is chemically similar to the bone mineral and has excellent biocompatibility with human bones [1, 2]. Porous HA scaffolds with high porosity and interconnectivity are intensively studied for applications such as skeletal defect fillers and bone tissue engineering [3, 4]. However, *in vivo* degradation of HA is slow, and this limits tissue ingrowth and scaffold replacement by new bone. Additionally, because of the simple chemistry of HA, these scaffolds do not possess properties desired for overcoming complex clinical problems, such as microbial infection and impaired bone regeneration in poor bone stock [5, 6]. Incorporation of polymeric drug-release carriers into HA scaffolds has been proposed as a solution to this limitation [7, 8]. Currently reported scaffold-carrier systems include coating scaffolds with polymer films [9], microspheres [10] and chemically grafted drugs [11].

The extracellular matrix (ECM) of human tissues commonly comprises a network of nano-sized proteinaceous fibers (e.g. collagen) and various biologically functional molecules such as cytokines. Cells bind to the ECM via surface receptors, whereby various cellular behaviors (e.g. migration, proliferation, differentiation) are activated. Accordingly, the ECM, its composition, structures and mechanic properties are inspiring for biomaterials design and fabrication [12, 13]. Emulating some characteristics of the ECM, e.g. by imparting a fibrous surface to a scaffold, may create a more favorable microenvironment for cells. Sun *et al.* [14] compared the biocompatibilies and osteogenic capabilities of four bone-related biomaterials (i.e. collagen, collagen/HA, HA, biphasic calcium phosphate) by culturing rat bone marrow stem cells (MSCs), and found that the cells on collagen/HA exhibited comparable upregulation in osteogenic gene makers to the osteogenic control group but had increased cell proliferation. Woo *et al.* [15] compared nanofibrous poly-l-lactide (PLLA) scaffolds with counterpart scaffolds having solid walls, and found the former had 4-fold higher serum protein adsorption and 70% higher osteoblast attachment. Smith *et al.* [16] cultured human embryonic stem cells on PLLA matrices with nanofibrous or traditional solid microstructures, and observed that the nanofibrous ones supported significantly higher osteoblastic gene expression and mineralization. Huang *et al.* [17] compared osteogenesis of sandwich-type PLLA-nanosheets with or without BMP-2 in mice, and found increased osteogenesis and new bone stiffness in 8 weeks in BMP-2-loaded nanosheets, whereas no bone formation occurred over a period of 20 weeks in BMP-2-free nanosheets. Su *et al.* [18] assessed the osteogenicity of collagen/HA in the absence or presence of recombinant human bone morphogenetic protein-2 (rhBMP-2) by cell culture and animal implantation. They observed that rhBMP-2 enhanced the bone formation and also expedited the degradation of CHA.

In light of these findings, we suggested that coating of scaffold surface with drug-laden fibers may create an ECM-like interfacial microenvironment and simultaneously allow drug release, providing duplex advantages. In the present study, we prepared porous HA scaffolds and coated their surfaces with short PLLA fibers containing bone morphogenetic protein-2 (BMP-2). It was found that, this modification simultaneously improved the compressive strength and osteogenesis of the scaffold, suggesting an effective approach to improving the properties of HA scaffolds.

## Materials and methods

### Fiber preparation

PLLA (0.8 g; Jinan Daigang Biomaterial Co., Ltd, China) was dissolved in 10 ml of a mixed solution (9 ml of dichloromethane and 1 ml of dimethylformamide). A BMP-2 solution (0.2 ml, 0.75 mg/ml; Pepro Tech, American) was added to the PLLA solution and stirred (10 000 r/min, 3 min) to form a water-in-oil emulsion. It was then electrospun to a fibrous mat (20 kV, 0.5 ml/h, mandrel rotation: 20 r/s; LSP01-1A, Longer Pump, China) and vacuum-dried overnight to remove residual solvent. The mat was cut with a customized apparatus to short fibers.

### Scaffold preparation

Porous HA scaffolds were prepared following our previous study [19]. Briefly, an HA slurry in chitin was forced to pass a mold packed with sucrose spheres, and subsequently immersed in water to rapidly induce chitin gelation and dissolve sucrose. The porous body thus formed was dried at 80°C overnight sintered at 1200°C for 2 h to form porous HA scaffolds. Samples used for mechanical testing were Φ8 × 12 mm cylinders, and those for all other experiments were Φ8 × 8 mm.

Sodium alginate solution is used as a dispersing agent to disperse short fibers. Pretests indicated that 2% sodium alginate produced optimal dispersion effect with minimal fiber agglomeration. Short PLLA fibers (0.5 mg) were dispersed in 10 ml of a sodium alginate solution (2%; w/w); after stirring (1200 r/m, 1 h), 10 ml of water was added to the suspension. Each scaffold was immersed in the suspension (scaffold/suspension = 0.1 g/ml), vacuumed for 15 min to encourage suspension infiltration to the pores, retrieved and centrifuged (2000 r/min, 60 s) to remove excess suspension. The scaffold was immersed in a 2% (w/w) calcium chloride solution for 5 min to crosslink alginate chains. Unless otherwise described, all reagents were purchased from Kelong Chemical (Chengdu, China), and double distilled water was used throughout the study.

### General characterizations

Sample morphology was studied by optical (Olympus BH-2) and scanning electron microscopies (JEOL JSM-7800F). Compressive strength was tested (0.5 mm/min; Instron 5567) using cylindrical scaffolds (Φ8 × 12 mm, *n* = 9). Total porosity was determined simply from the masses of samples (*n* = 30) and the theoretical density of HA, and effective porosity was measured following Archimedes principle by immersing each sample AQ11in 5 ml of water following a previous study (*n* = 30) [20].

For *in vitro* BMP-2 release from short fibers or fiber-coated scaffolds, each sample (6.6 mg of fibers or one fiber-coated scaffold) was immersed in 2 ml of phosphate buffer saline （PBS） (pH 7.2), maintained in an orbital water bath (37°C, 100 r/min). At predetermined intervals, 0.5 ml of liquid was collected for BMP-2 assay (Ray Biotech, Norcross, GA, USA), and the PBS was replenished.

### *In vitro* cell proliferation and alkaline phosphatase activity

Rat calvarial osteoblasts (RCOBs) and bone marrow stem cells (MSCs) were isolated from neonatal Sprague Dawley (SD) rats (10-day-old, male; Dashuo biotech). Briefly, RCOBs and BMSCs were harvested from calvarium and femora and tibiae of SD rats, respectively [21].

#### Proliferation

Four groups of scaffolds (*n* = 9/group; Φ8 mm × 8 mm) were used to evaluate *in vitro* cytocompatibility: (i) uncoated HA scaffolds (abbreviated as HAs), (ii) scaffolds coated with alginate alone (SA-HAs), (iii) scaffolds coated with PLLA fibers carrying no BMP-2 (F-HAs) and (iv) scaffolds coated with BMP-2-loaded PLLA fibers (BMP-HAs, 1.24 μg BMP-2/scaffold). All scaffolds were placed in culture plates and UV irradiated overnight. Each scaffold was seeded with RCOBs (1 × 10^4^ cells/sample) and incubated for 30 min to allow attachment. Then, 1 ml of α-MEM supplemented with 15% fetal bovine serum (FBS, Excel, China) was added to each well, and the samples were incubated with a medium change every 2 days. After incubation for 1, 3, 5 and 7 days, cell proliferation was evaluated by Alamar blue assays. The cell number was quantitated by measuring the optical density (OD) at 570 nm with a microplate reader (UQuant MQX200, Bio-Tek, Winooski, VT, USA), following routine procedures [22].

#### Alkaline phosphatase activity

Each sample was seeded with 2 × 10^4^ BMSCs and cultured in an induced osteogenic medium (α-MEM, 10% FBS, 1% penicillin/streptomycin, 10 mM β-glycerolphosphate, 50 mg/ml vitamin C) with a medium change every 2 days. After culture for 7, 12 and 21 days, alkaline phosphatase (ALP) activities were measured with ALP kits and BCA kits (Jiancheng Biotech, China). Briefly, each sample was removed, rinsed with PBS twice, treated with 1 ml of Triton X-100 (BioFROXX, China) (3 cycling treatments between −20°C and 25°C). The supernatant was collected; the OD at 520 nm was measured and normalized to the total protein content [23]. Each experiment contained nine parallel samples.

### *In vivo* osteogenesis

Osteogenesis of three groups (HAs, F-HAs, BMP-HAs) were evaluated in four male dogs (10–14 months, 12–16 kg; Sichuan Provincial Experimental Animal Center, Chengdu, China). After anesthetization (30 mg/kg intravenous pentobarbital), 12 incisions (T9–L1, length: 2 cm) were made 3 cm off the spine. The skin was elevated, and pockets were created in the underlying muscles by blunt separation. One sample was placed in each pocket, and the wound was sutured layer by layer. The dogs received intramuscular penicillin injection (803104IU) for 3 days to prevent infection. Each dog was caged separately and allowed free access to water and food. Four and twelve weeks after operation, the dogs were killed by pentobarbital overdosing. Samples were retrieved, fixed in formalin (10%, 3 days), decalcified (12.5% ethylenediamine tetraacetic acid, 60 days), dehydrated in ethanol series and mounted in paraffin. They were cut perpendicular to the longitudinal axis to sections (4–6 μm), and stained separately with hematoxylin–eosin and Masson trichrome reagent. Stained sections were studied under a light microscope for new bone formation and measured with Image-J program. All procedures were approved by the Research Ethics Committee of Southwest Jiaotong University.

### Statistical analysis

Data are presented as the mean ± standard deviation. Data were analyzed by analysis of variance (ANOVA, Prism 6.0, Graphpad, San Diego, CA, USA) and Tukey multiple comparison test. A *P* < 0.05 was considered statistically significant.

## Results

### Scaffold, PLLA fibers and BMP-2 release

**Figure 1:**
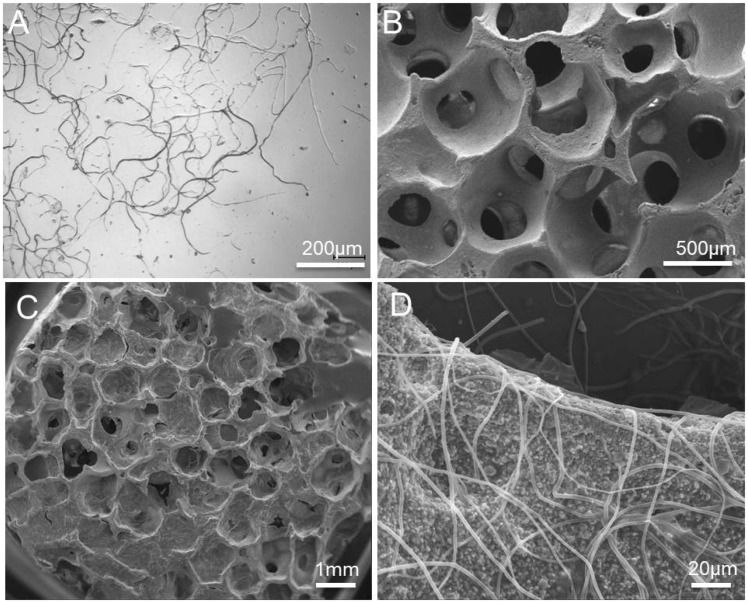
(**A**) Light micrographs of PLLA fibers; scanning electron micrographs of (**B**) uncoated HA scaffold and (**C** and **D**) scaffold coated with PLLA fibers

**Figure 2:**
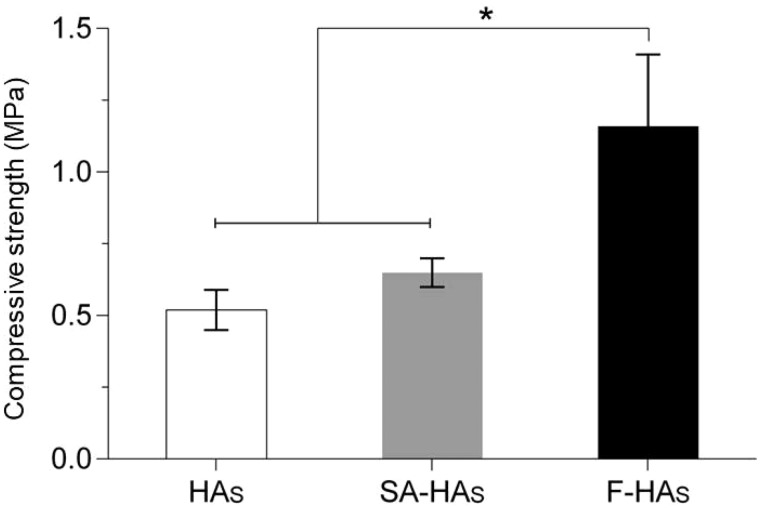
Compressive strengths of scaffolds (*n* = 9)

The short fibers were ∼400–600 μm long, ∼1–2 μm in diameter and well dispersed in 2% sodium alginate without severe aggregation (Fig. 1A). The uncoated HAs scaffold had a cancellous morphology with interconnected pores ∼500 μm in diameter (Fig. 1B). It had a total porosity of 79.2%, an effective porosity of 70.5% (Table 1) and a compressive strength of 0.52 ± 0.07 MPa. After dip coating-centrifugation (General characterizations section), PLLA fibers were uniformly deposited on the pore surface, forming a loose network (Fig. 1C and D). After coating, the total porosity and effective porosity decreased to 75.8% and 67.9%, however, compressive strength increased to 1.16 ± 0.25 MPa (Fig. 2).

**Table 1:** Porosity characteristics of HA scaffolds before and after coating

Sample	Porosity (%) (*n* = 30)	Effective porosity (%) (*n* = 30)
Has	79.2 ± 1.9	70.5 ± 2.3
SA-HAs	77.6 ± 2.6	69.3 ± 3.8
F-HAs	75.8 ± 2.9	67.9 ± 3.1

**Figure 3:**
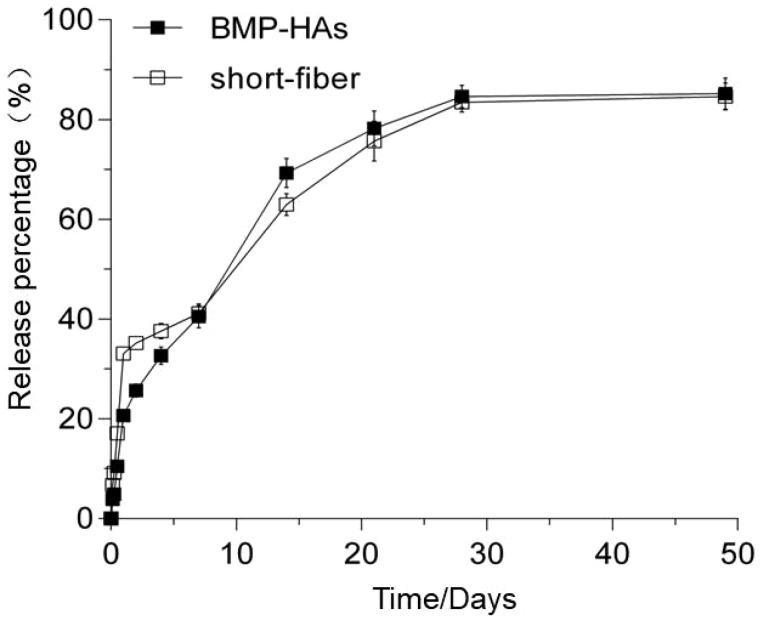
Cumulative release curves of BMP-2 from PLLA fibers dispersed in solution and fiber-coated HA scaffolds (BMP-HAs, all *n* = 9)

*In vitro* release tests found that the short fibers dispersed in the solution burst-released 33.0% of the BMP-2 load within Day 1, and subsequently almost linearly released 83.5% of the load till Day 28 (Fig. 3). After the coating procedure (Scaffold preparation section), 1.24 μg of BMP-2 was loaded onto each scaffold. The resultant BMP-HAs scaffold gave a lower burst initial release (20.7% in Day 1), followed by slower linear release (40.5%) till Day 7 and by a slightly further slower linearly release (87.6%) till Day 28 (Fig. 3).

### *In vitro* cell culture

#### Proliferation

**Figure 4:**
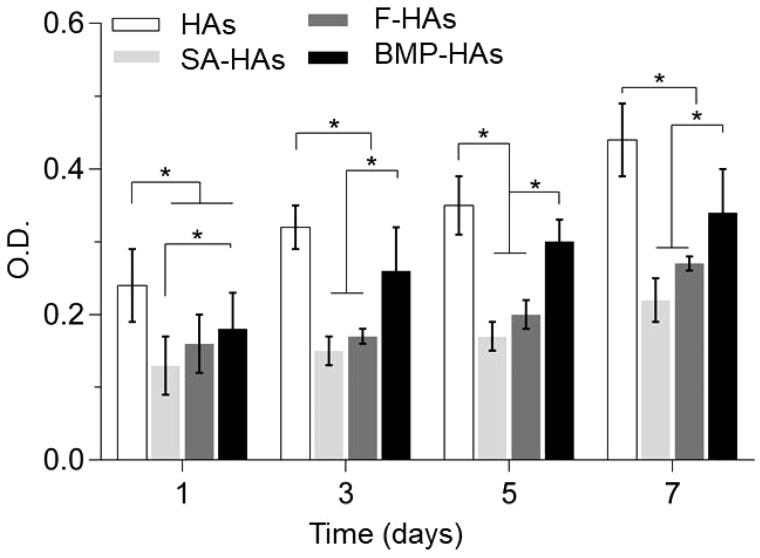
RCOBs proliferation after culture on various scaffolds for 1–7* *days (*P <* *0.05, *n* = 9)

Alamar blue assays found that RCOBs seeded on all scaffolds continued to proliferate between Days 1 and 7 (Fig. 4). Throughout this period, compared with HAs, the cell numbers on the other three scaffolds were significantly reduced (all *P* < 0.05) but they increased in the order of: SA-HAs < F-HAs < BMP-HAs. On Days 3, 5 and 7, the cell number on BMP-HAs was significantly lower than that on HAs but significantly higher than those on SA-HAs and F-HAs (all *P* < 0.05). Throughout the period, the difference between SA-HAs and F-HAs was not statistically significant (all *P* > 0.05, *n* = 9).

#### ALP activity

**Figure 5:**
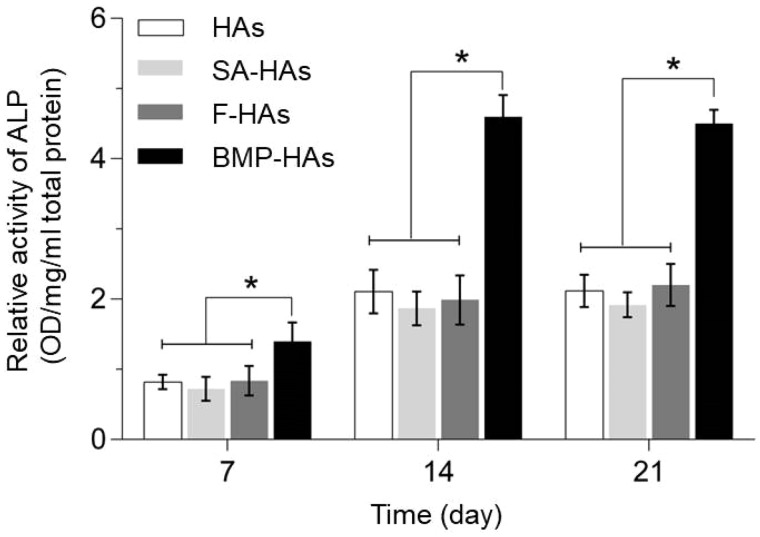
ALP activities of BMCSs after culture for 7, 14 and 21* *days (*P <* *0.05, *n* = 9)

ALP is an early marker of osteoblastic differentiation of MSCs. Therefore, after culture in the induced osteogenic medium for 7–21 days, ALP activities were measured and normalized to the total protein content. At each time point, the normalized ALP activity measured from BMP-HAs was significantly higher than those from the other three scaffolds (all *P* < 0.05), whereas the difference between any other pair was not statistically significant (all *P* > 0.05) (Fig. 5). After 21 days, compared with HAs, the normalized ALP activities measured from the other three scaffolds were 9.4% lower (SA-HAs), 3.8% higher (F-HAs) and 112.3% higher (BMP-HAs), respectively.

### *In vivo* performance

#### Osteogenesis

**Figure 6:**
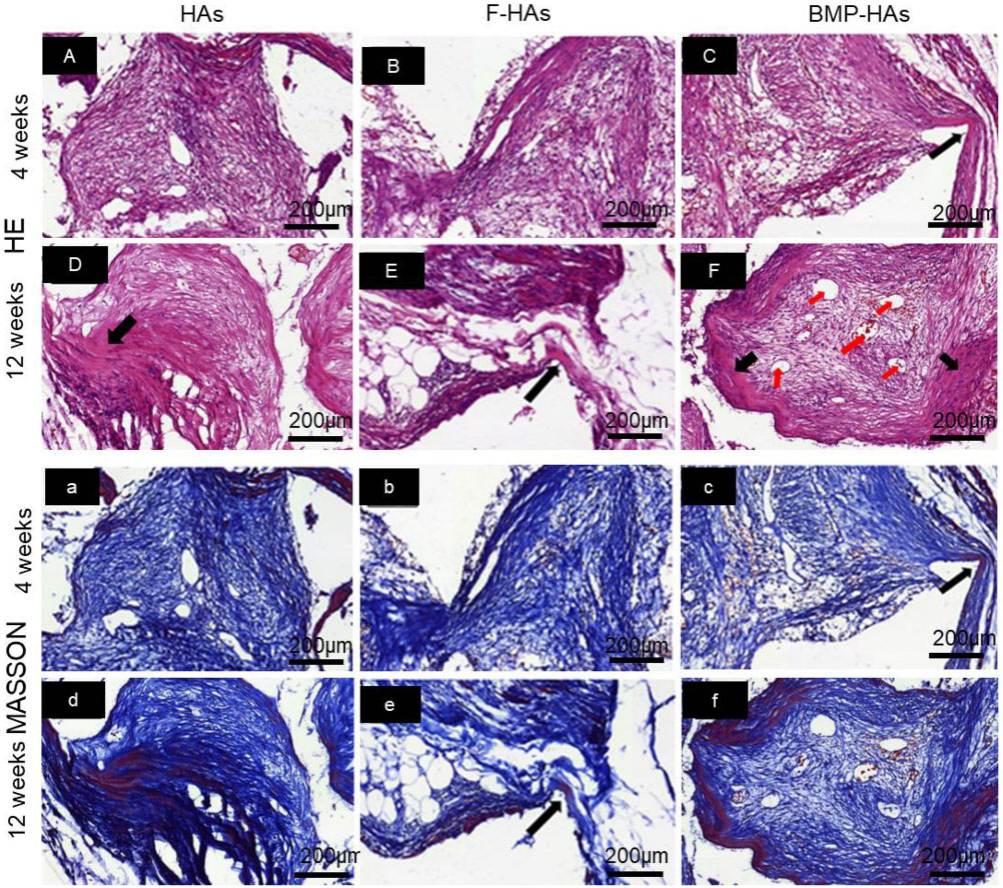
(**A**–**F**) HE and (**a**–**f**) Masson trichrome stained sections revealing soft tissue and new bone formation in various scaffolds; red arrowheads: new blood vessels, black arrowheads: new bone

**Figure 7:**
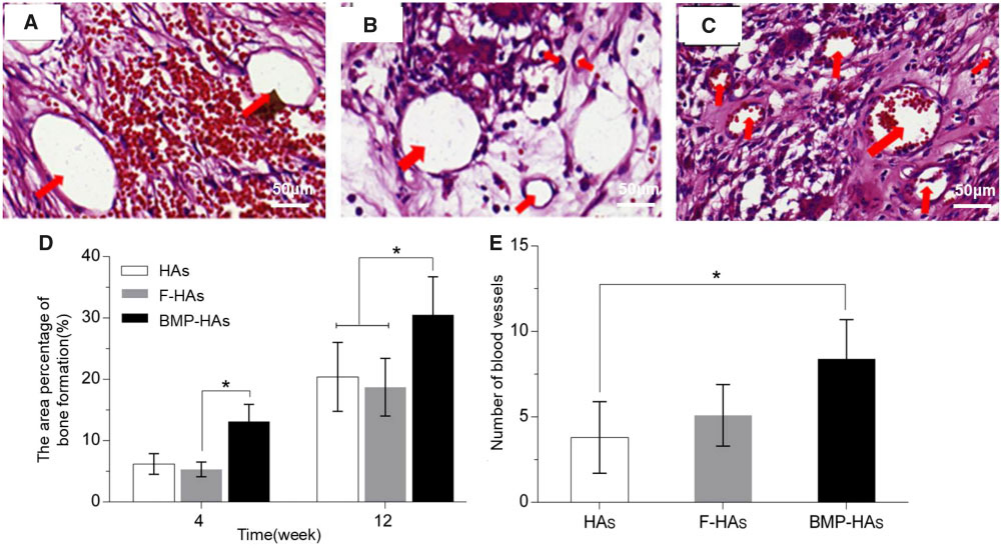
HE-stained sections revealing new vessels (red arrowheads), HAs (**A**); F-HAs (**B**); BMP-HAs (**C**); (**D**) new bone area fractions at Weeks 4 and 12 and (**E**) new blood vessel density (mm^−2^) at Week 4 (*n* = 4)

Scaffolds retrieved retained their structure without noticeable collapse or other deformations. HE and Masson trichrome staining revealed that, at Week 4, fibrous tissues and new bone penetrated into all three groups of scaffolds (Fig. 6A–F, a–f). The area of fibrous tissues followed the rank of: F-HAs > HAs > BMP-HAs. The new bone formed in BMP-HAs covered 13.1% of the defect area, compared with 5.3% in F-HAs and 6.2% in HAs (Fig. 7D); the differences between BMP-HAs and the other two groups were both statistically significant (all *P* < 0.05). The new bone in all groups appeared morphologically immature.

At Week 12, new bone areas in all groups increased (Fig. 6A–F, a–f). The area and maturity of new bone in F-HAs increased only marginally compared with Week 4. The new bone in BMP-HAs largely surrounded the pore surface and appeared relatively mature in morphology. The new bone area fraction in BMP-HAs (30.4%) was significantly higher than those in HAs (18.7%) and F-HAs (20.4%) (Fig. 7D).

HE staining also revealed that, at Week 4, mature new vessels formed in all groups (Fig. 7A–C), with densities of 3.8 vessels/mm^2^ in HAs, 5.1 vessels/mm^2^ in F-HAs and 9.4 vessels/mm^2^ in BMP-HAs (Fig. 7E). The new vessel density in BMP-HAs was statistically higher than those in the other two groups (both *P* < 0.05).

## Discussion

Results of the current study show that, coating of HA scaffolds with BMP-2-loaded PLLA fibers stimulated osteoblastic differentiation of MSCs, improved compressive strength of scaffolds and significantly increased osteogenesis and angiogenesis in canine muscles. Expectedly, these affects may contribute to better outcomes of HA scaffolds in clinical bone defect repair.

The short fibers were well dispersed on the scaffold surface without obstructing the pore interconnectivity (Fig. 1C–D), although they slightly reduced the porosity (Table 1). Compressive strength is a fundamental requirement for bioceramic scaffolds. Alginate coating alone improved the compressive strength marginally, because of the low tensile strength of alginate (Fig. 2) [24]. After fiber coating, the compressive strength increased substantially to 1.16 MPa, entering the lower range of human trabecular bone (i.e. 1–10 MPa). This is similar to another report that the introduction of polylactide-co-glycolide into porous β-TCP scaffolds effectively enhanced their mechanical properties [25]. Zhao *et al.* [26] reported that composite coatings significantly improved the mechanical and biological properties of HA scaffolds while retaining the 3D interconnected porous structure. The mechanical improvement was explained by a combination of the full coating of struts, the interpenetrating structural characteristics and crack bridging by the polymer. According to composite mechanics, the fibers probably bridged cracks to resist their opening during compression, thus improving the scaffold strength. The BMP-HAs yielded a lower initial burst release of BMP-2 than did the free fibers because the alginate layer provided an additional diffusion barrier (Fig. 3). The nearly linear release till Day 28 is appropriate for bone defect healing, as active callus formation usually occurs in the first 4 weeks.

The alginate coating significantly reduced the initial adhesion of RCOBs compared with HAs, but did not impair their proliferation (Fig. 4). This may be partly attributed to the surface features of the two scaffolds. HAs presented a rough surface and abundant micropores, supporting MSCs attachment. The alginate coating covered these surface features and presented a smooth surface unfavorable for cell attachment. The coating of drug-free PLLA fibers moderately increased cell adhesion, likely because of the surface roughness introduced. Coating of BMP-2-loaded fibers significantly improved the MSCs adhesion but it remained significantly lower than HAs throughout Days 1–7, likely because BMP-2 more effective affected MSCs differentiation (vs. proliferation). Recently, Zhang *et al.* [27] compared HA scaffolds carrying microspheres with or without BMP-2, and found that BMP-2 released promoted the differentiation of BMSCs and led to a slower proliferation. On Days 14 and 21, BMP-HAs produced approximately one-fold increase in ALP activity compared with all other groups (Fig. 5), strongly indicating that the released BMP-2 stimulated the osteoblastic differentiation of BMSCs seeded on the scaffolds. The other three groups differed moderately, suggesting that their surfaces had relatively minor impact on MSCs differentiation.

Several strategies have been reported for attaching drug microcarriers to porous scaffolds. Of these, immobilization of microspheres is a commonly used method. Compared with microspheres, fibers may offer a few advantages. Fibers are readily attached to a scaffold via mechanical wrapping-intertwining (Fig. 1D). In comparison, microspheres form point contacts with a scaffold and the attachment can thus be weak, resulting in potential detachment. Son *et al.* [10] electrostatically immobilized microspheres to HA scaffolds by precoating with polyethyleneimine (PEI). However, PEI has been reported to be cytotoxic. In comparison, mechanical attachment of fibers requires no potentially cytotoxic agents. Additionally, microspheres are commonly prepared via emulsion techniques, and are frequently associated with low drug encapsulation efficiencies due to drug partitioning between solvents. Zhang *et al.* [27] found that microspheres carried 110 ng of BMP-2 per 1 mg of microspheres, corresponding to encapsulation efficiency of 14.7%. Wei *et al.* [28] found that both BMP-2 distribution studies and entrapment efficiency studies demonstrated that soybean lecithin (SL)/BMP-2 complexes significantly increased the BMP-2 entrapment amount (from 25.1% to 71.8–83.3%) of microspheres. In comparison, electrospun fibers intrinsically features high encapsulation efficiencies as only one solvent is involved in electrospinning.

Osteogenesis in non-skeletal tissues, also known as osteoinduction, is a challenging model for evaluating the osteogenic properties of orthopedic materials [29–31].

Osteogenesis in such sites requires induction of uncommitted stem cells to osteoblasts, via physical or biochemical stimuli. At Week 4, F-HAs and HAs were primarily filled by soft tissues as they gave only weak stimuli (Fig. 6). A set of osteoinductive calcium phosphate bioceramics (CaPs) have been reported, the exact processes underlying osteoinduction, and the role of the physical and chemical properties of the ceramics, remain incompletely understood. Othman *et al.* [32] found that plasma cell glycoprotein 1 (PC-1), encoded by the ectonucleotide pyrophosphatase/phosphodiesterase 1 (ENPP1) gene, played a key role in osteoinduction by CaPs. Additionally, one theory proposes that CaPs adsorb, and thus concentrate, BMPs circulating in the body fluid and the adsorbed-concentrated BMPs subsequently triggers bone formation [33]. Cheng *et al.* [34] bilaterally implanted porous CaP implants into leg muscles of mice with unilateral fibular fracture, and observed that implants in the fractured side both formed new bone and did so earlier than the contralateral ones. Their findings indicate that the microenvironment (i.e. distance from site of fracture healing) strongly affects osteoinduction, supporting the adsorption-concentration theory. In the present study, BMP-HAs formed significantly more new bone than the other two groups at both Weeks 4 and 12. This is consistent with the BMP-adsorption theory and is also expected from the osteoinductive function of BMP-2. Significantly more blood vessels were detected in BMP-HAs than in the other two groups, as BMP-2 is also an angiogenic stimulant [35–37]. Duan *et al.* [38] recently reported that, addition of KRN633 (an inhibitor of angiogenesis) to osteoinductive CaPs significantly disrupted angiogenesis, delayed the onset of osteoinduction, and markedly reduced new bone area after implantation in canine dorsal muscles for 12 weeks. Their results highlight the critical role of angiogenesis in osteoinduction, consistent with the positive outcome observed in BMP-HAs observed in our study.

## Conclusion

Short PLLA fibers containing BMP-2 were coated on porous HA scaffolds to emulate the nanofibrous structure of ECM and simultaneously offer osteogenic substance release. The fibers affected porosity minimally but improved the compressive strength by 123%. The fiber-coated scaffolds yielded a burst of 21.5% in 1 day followed by a near-linear release for 24 days. The fiber-coated scaffolds had a lower *in vitro* RCOBs adhesion compared with the uncoated scaffold, but supported normal RCOBs proliferation. The coated scaffolds significantly increased the ALP activity of MSCs. After implantation in canine dorsal muscles, the coated scaffolds formed significantly more new bone at Weeks 4 and 12 and more blood vessels at Week 12 compared with the uncoated scaffolds and those coated with BMP-free fibers. Thus, fiber coating is an effective approach to simultaneously improve the mechanical and osteogenic properties of HA scaffolds. This new method of short fibers coated scaffolds provides a new option for drug delivery systems, furthermore, this approach may contribute to improved outcomes of clinical bone defect repair.
